# The Finite Element Method in Thermosetting Polymers’ and FRPs’ Supramolecular Structure and Thermomechanical Properties’ Modeling

**DOI:** 10.3390/polym16233443

**Published:** 2024-12-08

**Authors:** Alexander Korolev, Alexander Zadorin, Maxim Mishnev

**Affiliations:** Department of Building Construction and Structures, South Ural State University, Chelyabinsk 454080, Russia; aazadorin@susu.ru

**Keywords:** thermosetting polymer, cured polymer, fiber reinforced plastic (FRP), modulus of elasticity (MoE), finite element (FE), modeling, supramolecular structure

## Abstract

The object of research is cured thermosetting epoxy polymer and FRP on the base of the same polymer matrix. The purpose of this research is to develop the finite element (FE) method in the modeling of cured thermosetting polymers and FRPs to predict their mechanical and thermal properties. The structural mathematical modeling with subsequent computer FE modeling was performed. The results of FE modeling were compared with the experimental data of cured polymer’s and FRP’s tensile strength and deformations under mechanical load at different temperatures. The design of the polymer’s FE model was based on the tetrahedral supramolecular structure and then transformed into FRP’s model by integrating glass fiber rods. Using the structural density as the structure model’s parameter, the relative size and disposition of the finite elements were determined. The viscoelastic properties are set in the model by regulating the structural density and compressive/tensile properties of joints. The long-term plastic deformation and stress relaxation were determined as the result of the supramolecular structure’s inner shearing with the decrease of its structural density. The FE models of the cured epoxy polymer and FRP were developed, making it possible to predict short-term and long-term deformations under load with high accuracy considering the temperature factor.

## 1. Introduction

The computational prognosis and regulation of short- and long-term deformations of thermoset polymers and FRPs under mechanical and thermomechanical loads is still a relevant problem due to its high significance in the design of FRP structures. Most FRP structures that are exploited under thermomechanical load are the elements of aspiration or gas-exhausting systems. The combination of the mechanical and thermal stresses results in the complex stress-strain state of an FRP during long-term exploitation [1,2].

Mathematical modeling of mechanical and thermal properties of thermoset polymers and FRPs was the subject of much research. They have used different methods:

(1) Analytical modeling has determined the functional models, which were developed from the imitative Kelvin–Voight model [3,4,5] to models with relaxation core and graphic methods of deformations’ prediction [6,7,8];
(2) Structural modeling is presented in different research where the structure was modeled using computer programs. For example, designing the 3D molecular models according to the definite angles of polymer molecules’ bonds [9,10,11,12], molecular dynamic designing of polymer’s molecular structure and properties [13,14], computer modeling of filled polymers’ and FRPs’ multi-scale heterogeneous structures on the molecular and supramolecular level [15,16,17,18].

In recent research, the modeling combines functional and structural multi-scale methods [19,20,21,22] with thermal dependencies of mechanical properties. However, the prediction of the cured polymers’ and FRPs’ operational properties remains a relevant scientific problem, especially for structural models. They are required to determine the basic elements, their configuration and relative positions in the model, as well as those elements’ mechanical and thermal properties. If the basic element is determined as a molecule, molecular or supramolecular cluster, the structural model receives very different designs and math models.

The adequate structural model is the basis of an FE model because the basic structural element determines the configuration and properties of finite elements. The use of FE modeling can make it possible to easily and quickly predict the operational properties of polymers and FRPs. It is the reason why there is a regular interest in it [23,24,25,26].

The previous author’s research aimed to investigate the deformation and stress relaxation of polymers and FRPs under heating after long-term thermosetting and applying mechanical load with a determination of the Kelvin–Voight model’s parameters [27,28].

**Figure 1:**
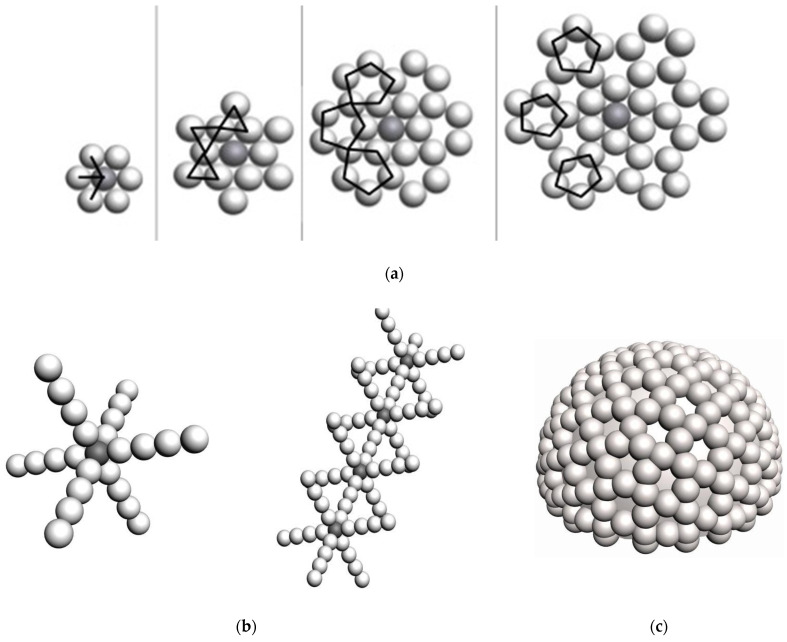
Models of formation of polymer’s supramolecular structure, (**a**)—core, (**b**)—axial, (**c**)—spherical.

The common conclusion of this research is that thermosetting polymer’s properties depend on the conglomerate layer or core structures. Therefore, the supramolecular structure belongs not only to the supramolecular polymers but to each homogenized thermosetting polymer. It is related to the supramolecular effect, presented in Figure 1.

The polymerization starts from the reactionary core. The supramolecular effect is concluded in the molecule pack’s density decreasing directly from the core to the periphery of the reactionary process. It results in the formation of micropores or leaky areas in supramolecular structures. It determines the parameters of the polymer’s and FRP’s supramolecular structural models, especially the structural density, and can be used in the FE modeling.

The FE modeling of FRPs’ inner structure requires considering the surface effect between the glass fiber and polymer. This effect results in the orientation of the polymer’s molecules towards the glass surface, increasing the density of the polymer in this area and forming the polymer shells. Due to that, the strength, elasticity and thermal expansion have a nonlinear increase with the growth of fiber concentration [23].

The purpose of this research is to design the structural model of cured thermoset polymer and FRP that determines their mechanical properties and parameters of an adequate FE model. To achieve this goal, the following investigations were performed:

- Experimental research of cured thermoset polymer’s deformations under the mechanical load in normal conditions and under heating, analysis of “stress-strain” curves to define the structural model;
- The design of an adequate FE-model of cured polymer’s deformations using the designed structural model and testing the model;
- The design of an adequate FE model of FRP using the polymer’s structural and FE models and testing the model.

As a result, new FE models of thermosetting polymer and FRP have been developed. Those models allowed us to accurately predict the stress-strain curve, modulus of elasticity and tensile strength of cured polymer and FRP under normal and elevated temperatures. The main novelties of the proposed method of polymer’s and FRPs’ FE modeling are as follows:

- Application of the supramolecular structural model of polymer in FE modeling;
- Introducing the glass fiber rods while considering the change in joint work between reinforcement and polymer matrix caused by the surface effect of polymer’s molecules reorienting in the contact zone with glass rods, resulting in the multi-scale model of FRP;
- The usage of FE models with the optimal number of elements provides accurate computation by a simple version of CAD programs that do not require a lot of memory and processor resources.

According to the investigations, the outline of the research includes the following:

1. Materials used in experimental research of cured polymer and FRP, methods of testing it to acquire thermomechanical properties, and structural and FE modeling.
2. Designing the supramolecular structural model of cured polymer with tetrahedral basic element and structural density parameter.
3. Determination of the finite elements in hinge-rod supramolecular structure of cured polymer and development of the algorithm of finite elements’ size characteristics’ calculation.
4. Results of experimental testing and FE modeling: the stress-strain curves, modulus of elasticity and tensile strength of the cured epoxy polymer.
5. Designing the structural model of the FRP under normal and elevated temperatures.
6. Results of experimental testing and FE modeling: the stress-strain curves, modulus of elasticity and tensile strength of the FRP.
7. Discussion and conclusion.

## 2. Materials and Methods

### 2.1. Materials

In this research, we studied the epoxy binder and the FRP on the base of the same epoxy binder and glass fabric T23. The same materials as in our previous research were used [27,28] (presented in Table 1).

The specimens were cured for 2 h at 80 °C and then, in case of thermal aging, kept for 24 h at 150 °C. This post-curing was conducted in order to change the specimen from an extremely plastic under-cured state closer to the real construction material.

### 2.2. Methods

#### 2.2.1. Testing

**Figure 2:**
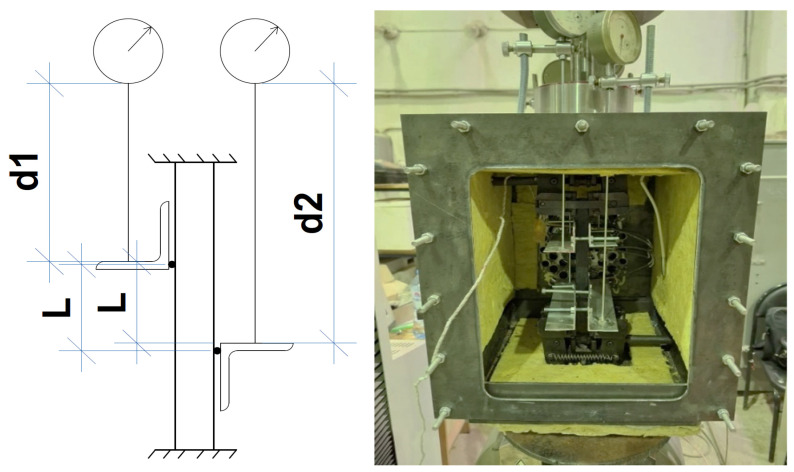
The test scheme (**left**) and the real photo (**right**).

The tests were carried out in a specially manufactured chamber that allowed the sample to be tested for tension by moving one of the chamber parts. Heating elements and a fan were installed in the chamber to create hot air movement and uniformity of heating inside the chamber. The chamber was installed on a Tinius Olsen h100ku machine (characteristics were presented in previous studies [27,28]). The upper part of the camera (see Figure 2) was clamped in the grip of the machine and was able to slip freely relative to the lower part. The strain of the sample was controlled by clock-type sensors to eliminate the effect of slippage in the grips of the machine and the test chamber. The test scheme and the photo of the real installation are presented in Figure 2.

#### 2.2.2. Modeling

**Figure 3:**
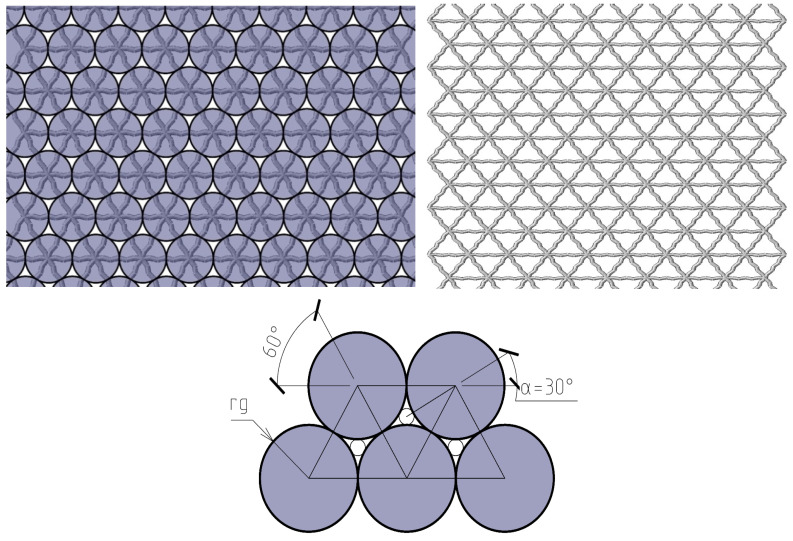
Dense (**left**) and not dense/leaky (**right**) molecular clusters cross-linking, geometric structure of dense globule lattice pack (**down**).

Simulation modeling of the supramolecular structure of the cured polymer was performed. Considering the need to specify the cross-linking of extended molecules and the angles of the cross-linking, flat and volumetric tetrahedral hinge-rod models were used as the basis. In such a model, the hinges are the cross-linking bonds, and the rods are extended molecules. In order to simulate structural changes in the polymer during prolonged loading or heating, models were developed from a dense to a leaky variant. The basic dense model (Figure 3, left) simulates the highly elastic properties of a cured polymer at the normal temperatures, assuming the thickness and length of the rod to be of the same order of size. This is an imitation representing the dense pack of molecules or molecular clusters in the cured structure. In the leaky model (Figure 3, right), a decrease in the ratio of thickness and length of the rod was assumed, resulting in the limited stability of the model with loss of elastic properties and strength.

**Figure 4:**
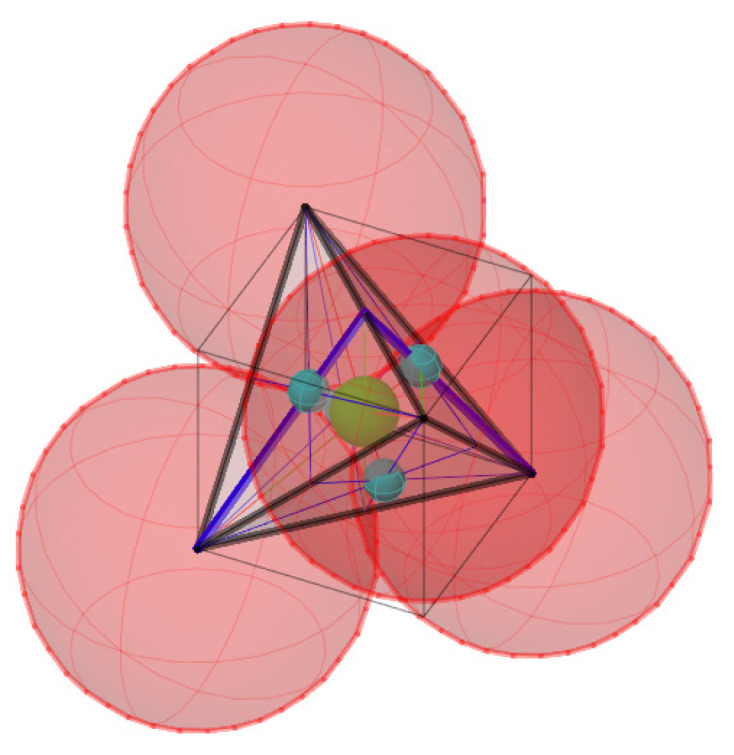
Basic supramolecular structural element of FE model.

**Figure 5:**
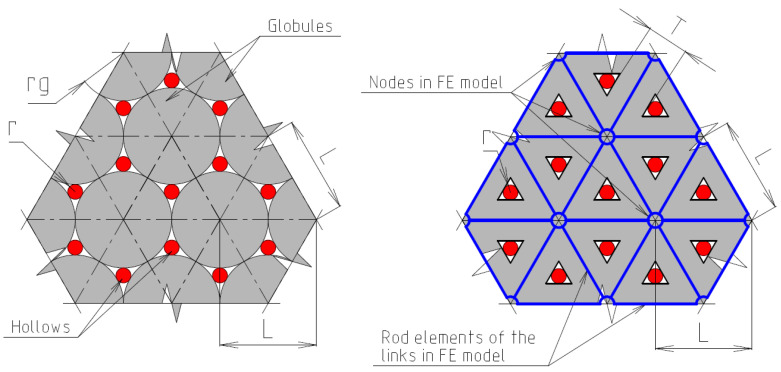
Transformation to hinge-rod model.

**Figure 6:**
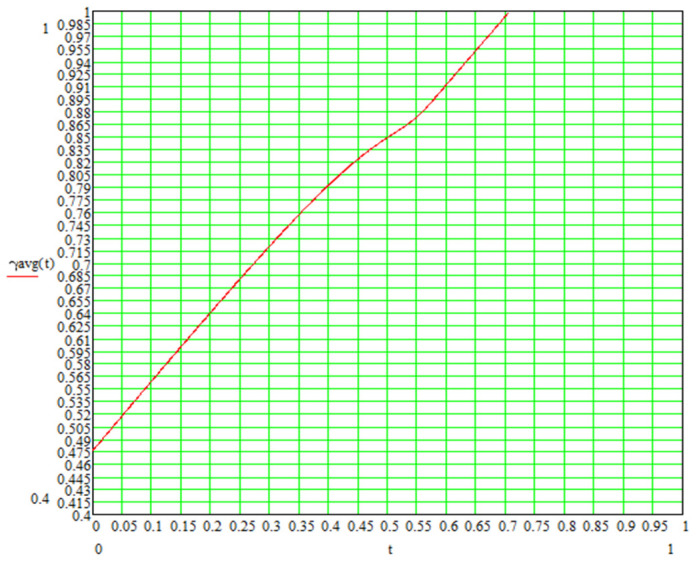
Relationship between structural density and relative size of joint in structural model.

Structural modeling for the purpose of predicting mechanical properties was implemented on the basis of the previously developed adequate author’s globule-lattice model (Figure 3, Figure 4, Figure 5 and Figure 6), as follows:

- The basic element is a polymerized globule;
- The discontinuity of the structure (the space is unfilled or filled with volatile, fragile groups) is a space trapped between globules;
- The structural model is a monofractional packing of globules. The degree of packing compaction determines the radius of the macrocapillary pores.

The basic analytical parameter in modeling is the structural density index, which was proposed earlier by the authors. It is expressed by the degree of compaction of cluster formations with definite radius. The structural density index realizes the relationship between volumetric porosity and linear size ratios of supramolecular formations:

(1) $$\gamma =\frac{r_{g}}{r+r_{g}}=cos\sqrt[{3}]{P}$$

P—volumetric macroporosity (discontinuity) is taken in radians in (1).

The structural density index was applied as a factor of the FE model. Finite element modeling was performed in the LIRA-CAD software package. The key parameter of the model is the thickness of the rod connections between the nodes (Figure 4). It was determined based on the structural density index, calculated by (1) for a given porosity. Transforming the (1) to (2):

(2) $$r=r_{g}\cdot \frac{1-\gamma }{\gamma }$$

From the analysis of Figure 3 of a dense package, the basic element is an equilateral triangle in the flat scheme and an equilateral tetrahedron in the volume scheme (Figure 4).

The discontinuity can be conventionally represented as spherical voids on the faces and in the center of the tetrahedron. However, the main volume is the central pore. The definition of the thickness of the rods is based on the condition of keeping constant porosity (see Figure 5).

As a result of the mathematical analysis of the presented model, formulas for determining the thickness of rods T in the model, where L is the length of the bond between globules, are obtained and given in Table 2.

The porosity is different on different planes and is considered as a plane-weighted average. Based on the obtained dependencies, a graph of the dependence of the structural density on the bond thickness at L=1 is constructed in Mathcad (Figure 6). Furthermore, the bond thickness is defined as the relative thickness δ=T/L.

**Figure 7:**
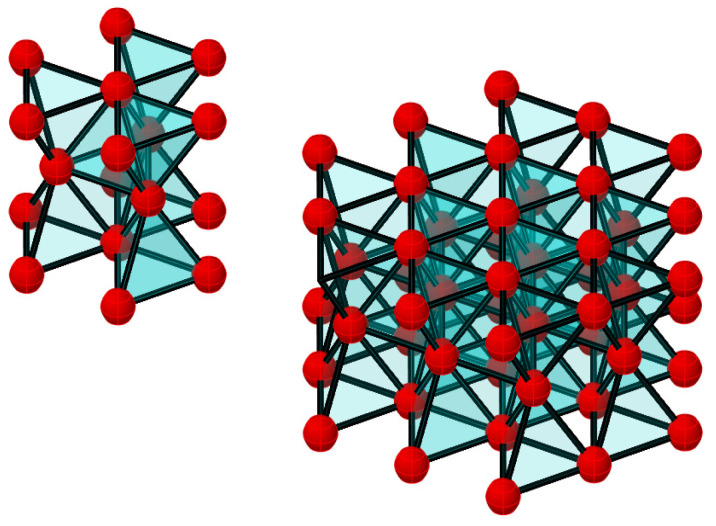
Three-dimensional scheme of FE model (scale is changed for presentation).

As a result, the FE model of the internal structure of the cured polymer was created from the basic tetrahedral elements. Part of the structure in the approximation is shown in Figure 7. The mesh is uniform and uniform throughout the volume, except for micro-roughness on the outer surface. At the same time, the structure is self-similar and allows us to build a model in any desired number of elements per unit volume.

**Figure 8:**
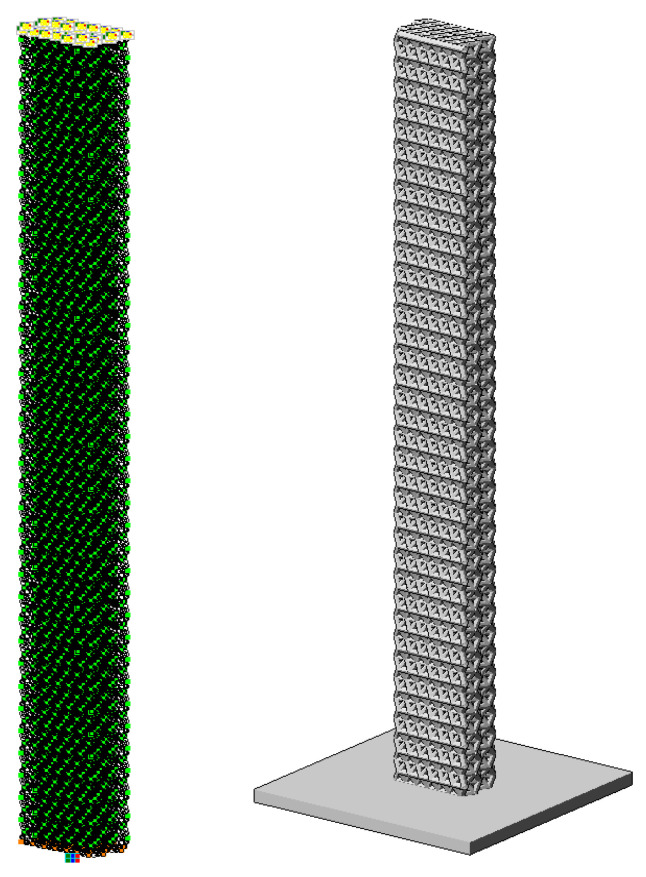
FE model in LIRA CAD.

The finite element model created in the LIRA-CAD software package is shown in Figure 8. The FE model was created with a similar aspect ratio to the tests performed (the sample sizes averaged about 20 × 2 × 1 cm).

The upper and lower faces were secured from transverse displacements (imitation of clamps on the scale of the sample and clamping by particles on the scale of the microstructure). Thus, the test scheme was simulated: the load was applied either in the form of a predetermined displacement of the upper nodes, i.e., a known relative deformation, or in the form of a force. The corresponding stresses were calculated as the longitudinal force divided by the area of the structure. The longitudinal force was collected as the sum of the reactions of the support nodes (it was controlled by a rod of high rigidity connected in series with the support nodes through a rigid body. The longitudinal force in the rod is equal to the force in the prism, and its deformations are zero. At each stage of the nonlinear calculation, deformation and force can be determined. Accordingly, it is possible to recalculate the data and obtain stresses, relative deformations and modulus of elasticity.

## 3. Results

### 3.1. The Study of Stress-Strain Curves and the Development of an Elastic FE Model of a Cured Polymer

**Figure 9:**
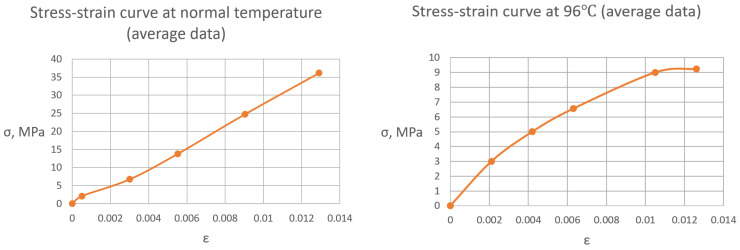
Stress-strain curves of glassed polymer under normal temperature (**left**) and 96 °C (**right**).

Figure 9 shows the stress-strain curves of the cured polymer samples at the normal temperature and at a temperature of 96 °C. It can be seen that the cured polymer has various deformative properties depending on temperature. At the normal temperature, the polymer works perfectly elastically with a sharp fracture when strength is achieved. In the heated state, plastic deformations develop from the beginning of loading, and the tensile strength decreases by an order of magnitude with no clear point of fracture and significant plastic area.

**Figure 10:**
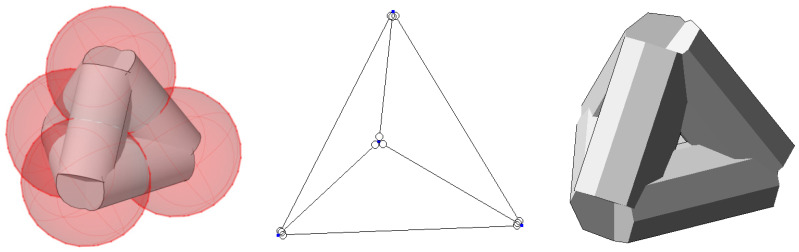
Basic hinge-rod element in FE model (tetrahedral with hinge joints).

In this regard, it was decided to develop a dense stable as a “cold” elastic model and a leaky unstable tetrahedral hinge-rod FE model as a “hot” plastic model, expressing plastic deformations as the development of the process of loss of local stability under loading. An analysis of the presented structural model was performed to determine the finite elements. It showed that each supramolecular element is a rod that is connected by hinges with other elements—also rods. Thus, the FE model of the polymer can be represented by a hinge-rod frame, the stability of which is determined by the location of the bonds/rods (Figure 10). Accordingly, the elastic deformation of the frame is the result of the elastic work of the rods under stress.

As a result, a volumetric tetrahedral model of the maximum degree of packing was created, in which the thickness of the bonds was determined by the structural density index according to formulas (1) and (2) and the graph in Figure 4. In order to start modeling, the constancy of the ratio of the modulus of elasticity and strength was established as a basic postulate. Table 3 shows the results of the correlation of elastic FE models of the polymer with the actual values of the modulus of elasticity.

**Figure 11:**
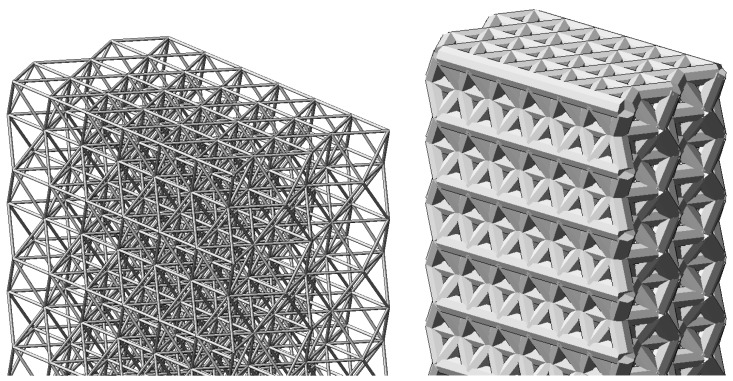
Three-dimensional model with T=0.05L (**left**) and T=0.54L (**right**).

From the above data, it can be seen that low bond thicknesses (Figure 11) lead to a sharp decrease in the elasticity of the model, which requires overestimating the elasticity of the bonds to an untrue level (right column in Table 3).

When calculating a model with different ratios of stiffness and bond strength, the constancy of the modulus and strength ratios of the model and bonds was revealed:

(3) $$E_{p}=k\cdot R_{p}$$

(4) $$E_{r}=m\cdot R_{r}$$

(5) $$\frac{k}{m}\approx 0.61\ldots 0.64$$

$E_{p},R_{p}$—modulus of elasticity and tensile strength of polymer;

$E_{r},R_{r}$—modulus of elasticity and tensile strength of rods;

k,m—correlation coefficients.

Therefore, knowing the actual ratio of stiffness and strength of the material from experiments, it is possible to determine the required ratio of stiffness and bond strength using the ratio $\left(5\right)$.

Polymers’ properties were known from previous experiments: $E_{p}\approx 3000\;MPa$ and $R_{p}\approx 38\;MPa$.

(6) $$k=\frac{E_{p}}{R_{p}}=\frac{3000}{38}\approx 79$$

(7) $$m=\frac{E_{r}}{R_{r}}\approx 130$$

**Figure 12:**
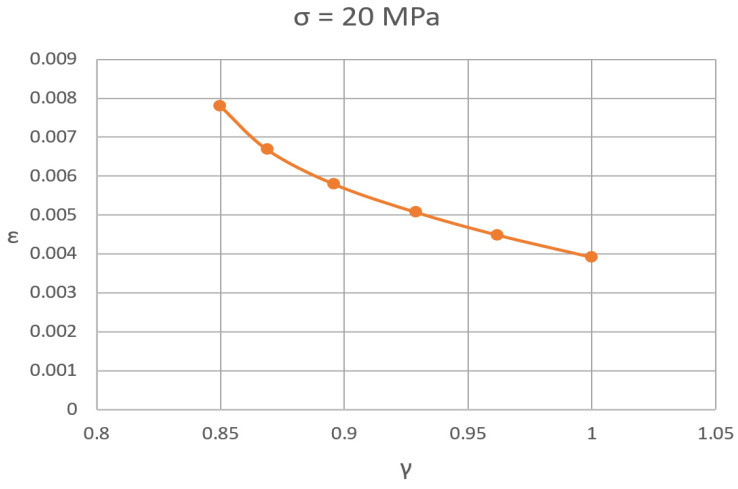
“Relative tensile deformation—FE model structural density “ curve.

At the same time, the absolute value of $E_{r}$ and $R_{r}$ is unknown. Thus, two unknowns remain for the final model—the absolute values of the stiffness and strength of the bonds and their thickness. It was not possible to determine the properties of molecular bonds; therefore, in order to build an adequate FE model, it is necessary to determine an adequate structural density of the model. According to the author’s research [22], during prolonged heating, the epoxy polymer loses up to 6% of the initial mass, which indicates that in the cold state, it contains volatile groups up to 8...9% of the volume, which corresponds to a structural density of 0.85...0.9 and a relative bond thickness of 0.5...0.6. These data allowed us to narrow the range of relative thickness of bonds in the FE model. The results of calculations of deformation at a given tensile stress of 20 MPa showed that the regulation of deformative properties in the FE model of the polymer occurs logically: an increase in structural density leads to an increase in bond thickness, resulting an increase in stability and a decrease in deformability (Figure 12).

The results of the structural density-adjusted correlation are shown in Table 4.

**Figure 13:**
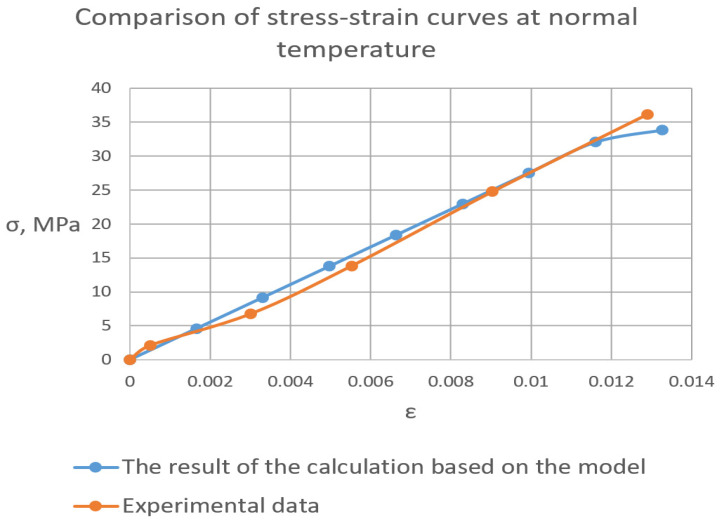
“Relative tensile deformation—tensile stress” experimental and FE model curves of cured polymer under normal temperature.

As a result, an elastic FE model was obtained, providing high convergence with the elastic properties of the cured polymer (Figure 13). For an epoxy material, the FE model with a structural density of 0.869 is adequate (convergence of more than 99%).

**Figure 14:**
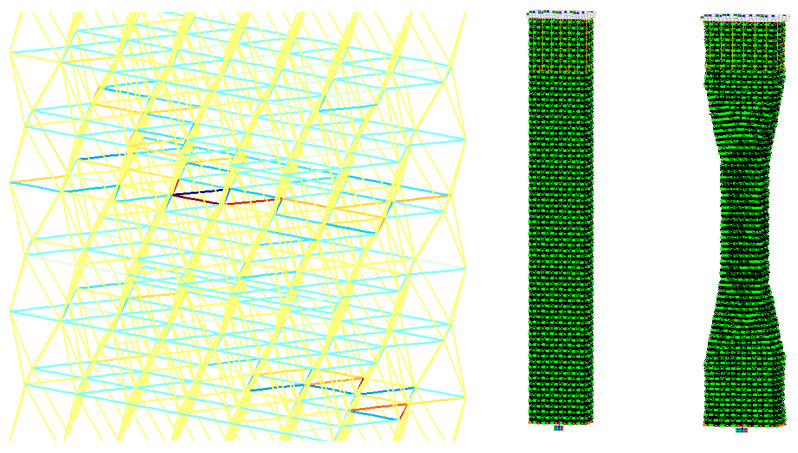
Loss of stability in compressed bonds (on the **left**). The shape (on the **right**) of the destruction (the scale of deformations has been increased for clarity).

For the FE modeling of a cured polymer at elevated temperatures, a leaky tetrahedral package was used, in which the strength of the rods and the modulus of elasticity/strength ratio correspond to the parameters of a dense package, and the thickness of the bonds is significantly less, which simulates a decrease in the number of cross-linking when they are heated and loss of elasticity. The calculation was carried out according to the model described above, considering geometric nonlinearity. As a result, in the heated state, compressed bonds lose stability under low loads, and plastic deformations occur in the model (Figure 14).

Table 5 shows the correlation data of the FE model of polymer deformation in the heated state at 96 °C. The actual values of the modulus of elasticity and strength at such a temperature are, on average, 1500 MPa and 9 MPa, respectively. According to the model, combinations of thicknesses and bond properties corresponding to such values are selected. Based on previous modeling experience, in order to increase the proportion of plastic deformations under loading, the difference in mechanical properties of bonds under compression and tension was set. This mention was based on the hypothesis that lowering compressive strength is the result of lowering the buckling of the rods in the structure under the heating. The mechanism has the next sequence: a decrease of the modulus of elasticity of the rods under the heating results in the increase of their flexibility with the lowering of the buckling. The reduced compressive strength is due to reduced critical stresses of the rod with lower buckling.

To specify in the model a decrease in the stability of heated bonds under compression, the compressive strength of the bonds is assumed to be an order of magnitude less than the tensile strength. Determining the exact value requires further research of the structural density or bond properties at elevated temperatures.

As a result, these correlations showed that the ratio of the modulus of elasticity and the tensile strength of bonds remained constant. The decrease in elastic properties is determined by a decrease in the structural density and thickness of bonds in the supramolecular structure. The proportion of plastic deformations is regulated by the ratio of tensile and compressive strengths.

**Figure 15:**
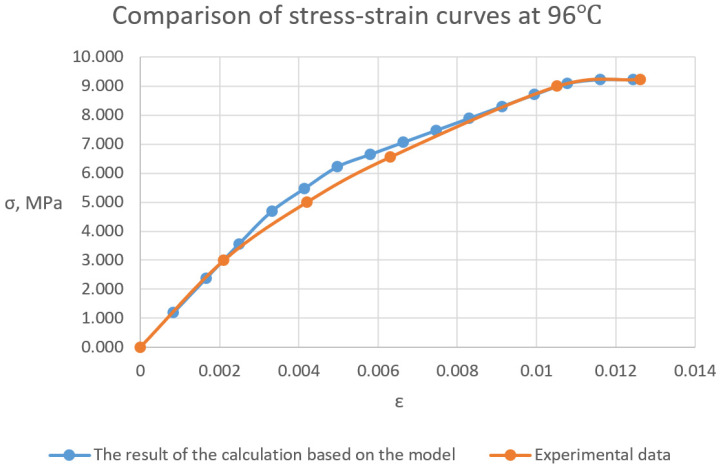
Experimental and calculated diagrams of deformation under load of a glassed polymer at a temperature of 96 °C according to the FE model.

**Figure 16:**
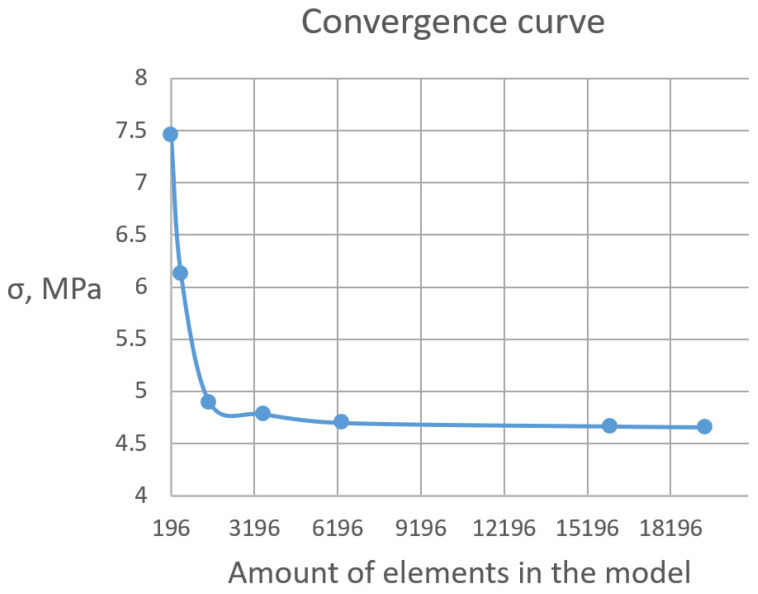
Convergence curve.

As can be seen from the comparative data of the graphs of the cured polymer obtained experimentally and accordingly to the model (Figure 15), the resulting FE model with a number of elements more than 18,000 shows a high level of convergence. The mesh convergence was validated through the assessment of normal stress in specimens at different levels of mesh refinement. The convergence curve of a normal stress is presented in Figure 16. Starting at the order of $10^{4}$ elements, convergence was achieved.

### 3.2. The Study of Stress-Strain Curves and the Development of an Elastic FE Model of a FRP

The behavior of FRPs under axial loads is linear elastic as it is determined by glass fibers. The properties of FRP strongly depend on the binder and reinforcement. Therefore, the experimental data and the model were based on the same composition: EP and T23 fiberglass. Strength and modulus of elasticity were obtained previously using our installation (see Section 2.2) and are presented in Table 6.

**Figure 17:**
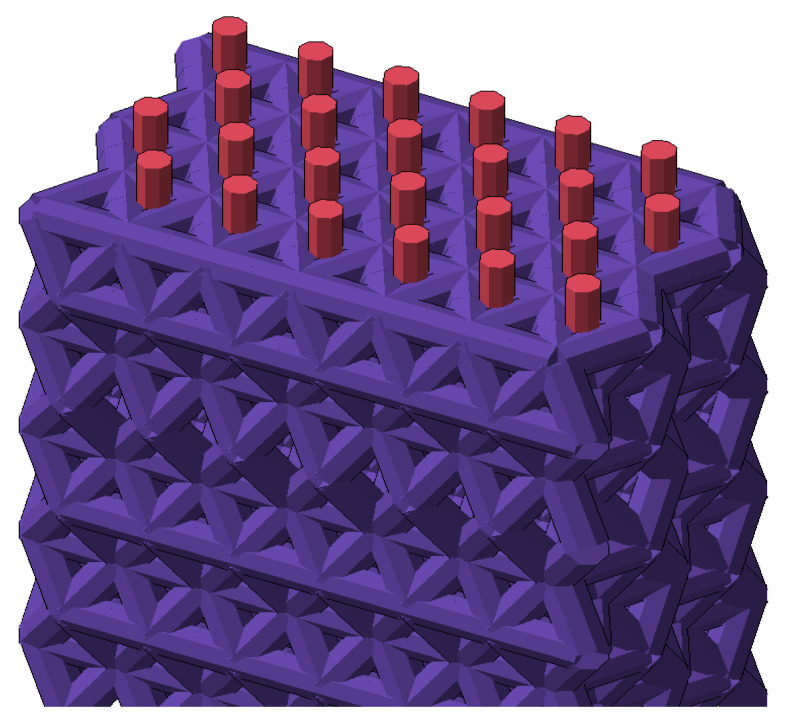
The section of FE model of FRP in 3D.

The FE model of an FRP was based on the FE model of a cured polymer (see Section 3.1). As was stated earlier, the structure of the model is self-similar and we do not build it using the real size and amount of links. The model of a polymer approximates a real chaotic structure, averaged based on the condition of matching the bond density per unit volume and matching the properties of the material. Using the same principle, the FRP model was developed by introducing the right proportion of rods that represent glass fibers. The amount of these elements and their size were determined by fiber volume fraction, which represents the area of glass fiber per unit of sectional area. Fiber volume fraction for similar material was obtained in previous work using micrographs [25]. Consequently, glass fiber rods were placed where the nodes of the FE model allowed, and their diameter was defined by the required fraction. Overall, the model is similar to the one in 3.2. The 3D view of the model with added fibers is shown in Figure 17. T23 fiberglass is made of E-glass with an average modulus of elasticity of 80–81 GPa and a strength of 3000–3500 MPa. In the current model, we used a modulus of 80 GPa and a strength of 3300 MPa.

**Figure 18:**
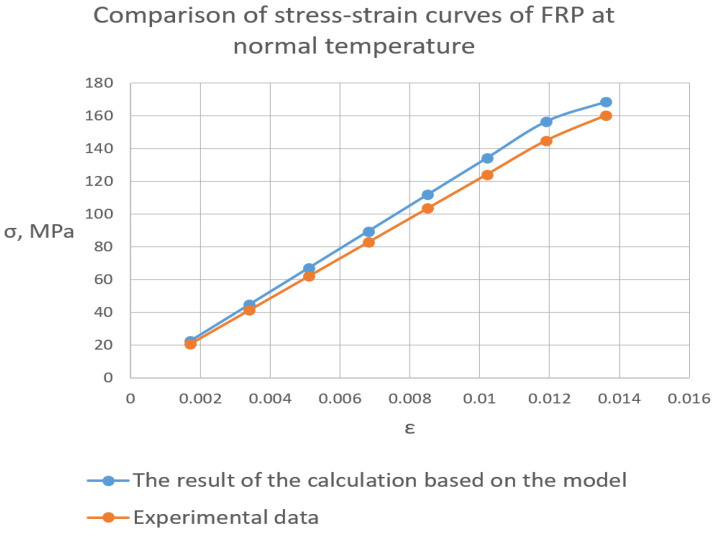
Experimental and calculated diagrams of deformation under load of FRP at normal temperature.

The model worked well at the normal temperature. There was an attempt to calculate at 96 °C by changing the properties of polymer links according to the data in Section 3.1. However, the modulus of elasticity and strength were higher than they should be, and the model requires improvement in further research. The stress-strain curves are shown in Figure 18.

Presumably, the discrepancy at elevated temperatures is caused by the need to consider changes in the joint work of the fiber and polymer, which weaken the material and do not allow the fiber to be fully included in the work. By analogy with how we reduced the thickness of polymer bonds in the polymer model, an attempt was made to simulate this weakening by reducing the proportion of fiber in the cross-section and introducing a reinforcement coefficient K. K is the ratio between the reduced fiber area and a normal one. In Table 7, this coefficient and the comparison of calculated and real elastic properties are presented. The estimated fraction of fibers in the section is presented in Figure 18. The fraction at the normal temperature, as was earlier stated, was obtained from real micrographs. The fractions at elevated temperatures were calculated using K. The results demonstrate a satisfying correlation.

**Figure 19:**
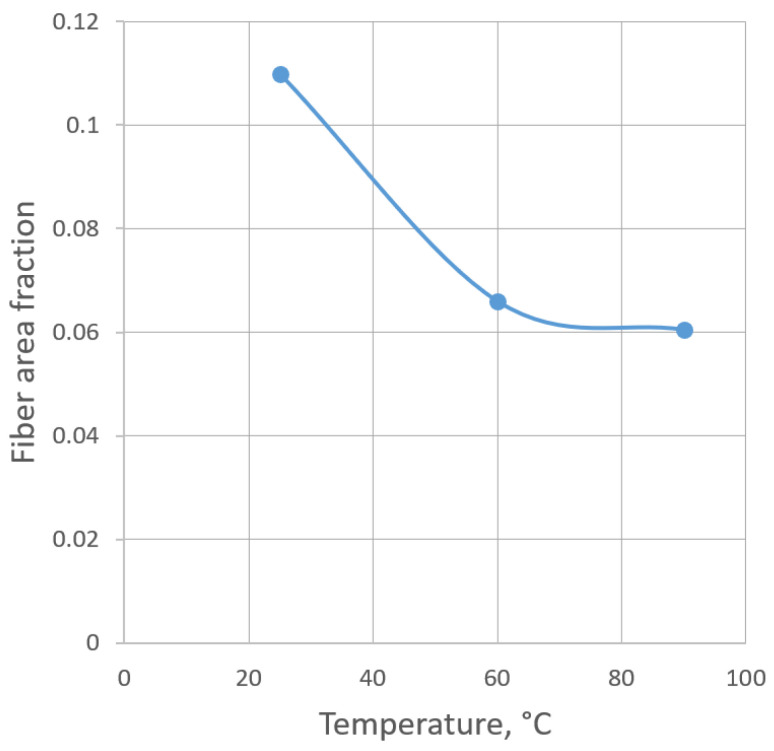
Fiber area fraction at different temperatures.

Fiber area fraction (Figure 19), determined as the ratio between the reinforcement’s total area in the cross-section and the area of the cross-section, shows that the fraction is reducing under heating. So, if the number of fibers is constant by the model, the index K is equal to the ratio of fibers’ diameter in the second degree after and before the heating. The ratio of the diameters before and after heating is equal to the correlation coefficient $k_{c}$ [29]:

(8) $$k_{c}=\frac{1}{\sqrt[{2}]{K}}$$

The $k_{c}$ is 1.29 under 60 °C and 1.35 under 96 °C. In the research [26] on FRP’s CTE, the $k_{c}$ depends on the glass fiber type, and for the glass fiber, T23 is determined as 1.27, which accords with the model’s parameters, taking into account some experimental variation of reinforcement’s concentration.

The results of testing have proved that the FE model of FRP is applicable for elevated temperatures as well, considering the weakened involvement of fibers into work, caused by surface effect. The “Cold” model contains the reinforcement rods with dense shells of the oriented polymer, and the “hot” model contains reinforcement rods with reduced cross-section because of the reorienting of the polymer shells under heating and weakened joint work between polymer matrix and glass fibers.

## 4. Discussion

As a result of this work, a simulation of the supramolecular structure of the cured polymer was performed, which makes it possible to predict its mechanical and thermal properties. Such supramolecular structural models were developed into the FE model of cured polymer and, further, of FRP. It makes it possible to predict promptly the exploitation properties of the polymer material.

The following principles of modeling a cured polymer were substantiated:

- The adequacy of tetrahedral hinge-rod structural models in the FE modeling of a cured polymer, in which the hinge is a basic globule molecular cluster, and the rods are the bonds between clusters;
- The disruption of bonds and the shift between clusters in the package is the cause of irreversible plastic deformations, creep and relaxation;
- The use of the structural density index as the main parameter of the models determines the relative thickness of the bonds in the model according to the author’s dependencies and, as a result, local stability and the proportion of elastic and plastic deformations under loading.

The parameters of adequate FE models of the cured polymer were determined as follows:

- The constancy of the relationship between the modulus of elasticity and the tensile strength of the bonds in the model;
- At the normal temperature, the structural density index is 0.85...0.86 with a relative bond thickness of 0.5...0.6;
- At elevated temperatures, it is necessary to reduce the compressive strength relative to tension and the structural density to match the actual deformation pattern. For example, at 96 °C, the structural density index of an adequate model is 0.72 with a relative bond thickness of 0.37.

The approbation showed that the developed FE models make it possible to predict stress, deformations and the modulus of elasticity of the cured polymer with high accuracy. The FE model allows for modification of the structure by adjusting the arrangement of elements, angles and connections between them or introducing components with modified stiffness of hinges or connections.

The major problem in FRP modeling is considering the joint work of fibers and polymer matrix. In the current model, the contact between fiber rods and polymer links is perfect without modeling friction or shear. As the temperature rises, the matrix becomes weaker, and these effects become more noticeable. That is why, in the initial model, the result at the normal temperature was satisfying, and the model failed at elevated temperatures. In the current work is proposed to reduce the involvement of the fiber in the work of the material (due to the weakening of the joint work with the matrix) by reducing the calculated area of the fiber in cross-section. By approximation, the reinforcement coefficient at different temperatures was determined, and with it, the model began to show an adequate result under elevated temperatures.

The proposed model of the cured polymer was successfully used to calculate the FRP based on that polymer at the normal temperature and under heating. Calculations at elevated temperatures required additional changes in the model, which were related to the change in the involvement of the fiber in the work of the material. Oriented polymer dense shells on the glass’ surface lose their density and orientation under the heating.

The principles of FE modeling of FRP are as follows:

- The glass rods in the model’s cells keep the scale and the ratio of glass fibers’ and polymer’s area fractions according to the real composition.
- At elevated temperatures, the base FE model has to be modified by reducing the reinforcement’s cross-section according to the correlation coefficient $k_{c}$ = 1.2...1.3.

In future work, it is planned to develop the model and consider this effect in a more appropriate way. For example, to introduce intermediate sections at the ends of the polymer rods adjacent to the fiber and simulate a malleable contact zone in those areas.

However, the current method of FE modeling already shows satisfying results. At the same time, it can be realized in any simple CAD program and does not require a lot of computational resources. Therefore, it can be used by any student, engineer or scientist in their research, study or work.

## Figures and Tables

**Table 1:** Types of binders investigated.

№	Composition	Name
1	Epoxy (Ker 828 52.5% + MTHPA 44.5% + alkofen 3%) (without thermal aging)	EP
2	Epoxy (Ker 828 52.5% + MTHPA 44.5% + alkofen 3%) (thermally-aged)	EP-TR
3	EP 46% + T23 54% (without thermal aging)	EP-T23
4	EP 46% + T23 54% (thermally-aged)	EP-T23-TR

**Table 2:** The joints’ thickness equations.

Pore Location	The Rod Joints’ Thickness Equations
Face	T=0.577L(2γ−1)
Centre	T=L(1.226γ−0.519)

**Table 3:** The various results of calculating the tensile strength and modulus of elasticity of a cured polymer according to the FE model for a given ratio of modulus of elasticity and bond strength.

Relative Thickness δ (in Relation with L)	0.54	0.27	0.2	0.1	0.05	0.05
E rod, MPa	5789	5789	5789	5789	5789	675229
R rod, MPa	44	44	44	44	44	5132
E polymer, MPa	2996	749	410	103	26	2996
R polymer, MPa	38	9.5	5.2	1.3	0.3	38
E polymer/R polymer	78.84211	78.84211	78.84615	79.23077	86.66667	78.84211
E rod/R rod	131.56818	131.56818	131.56818	131.56818	131.56818	131.57228
γ	0.869	0.697	0.641	0.559	0.518	0.518
$P={\left[arcos\left(\gamma \right)\right]}^{3}$	0.1387	0.5112	0.6699	0.9343	1.0809	1.0809

**Table 4:** The results of calculating the tensile modulus of elasticity of a cured polymer according to the structural density’s diapason.

Relative Thickness δ (in Relation with L)	0.5	0.54	0.58	0.62	0.66	0.7065
**γ**	0.85	0.869	0.896	0.929	0.962	1
$P={\left[arcos\left(\gamma \right)\right]}^{3}$	0.1708	0.1387	0.0974	0.0545	0.0212	0.0000
σ = 20 MPa						
ε	0.007802	0.0066779	0.0057886	0.0050671	0.0044799	0.0039094
Modulus of deformation, MPa	2563	2995	3455	3947	4464	5116

**Table 5:** Calculation data for the FE model of the modulus of elasticity and compressive strength of an epoxy-cured polymer at a temperature of 96 °C.

Temperature	Normal	96 °C				
Er	5778	fact	5778	4812	4000	3000
Rr tensile	44		44	36.6	30.4	22.8
Rr compress	44		4.4	3.66	3.04	2.28
tr	0.516		0.37	0.406	0.445	0.516
E polymer	2800	≈1500	1424	1424	1422	1434
R polymer	34	≈9	9.165	9.165	9.152	9.23

**Table 6:** Experimental and calculated data of FRP.

	Experimental Data		Calculated Data	
Temperature	E, MPa	R tensile, MPa	E, MPa	R tensile, MPa
Normal (25 °C)	12,000	160	13,000	168

**Table 7:** Experimental and calculated data of FRP at different temperatures.

	Experimental Data	Calculated Data	K
Temperature	E, MPa	E, MPa	
Normal (25 °C)	12,000	13,000	1
60 °C	9000	9060	0.6
96 °C	7300	7265	0.55

## Data Availability

The original contributions presented in this study are included in the article material. Further inquiries can be directed to the corresponding authors.
